# Evaluating gentrification’s relation to neighborhood and city health

**DOI:** 10.1371/journal.pone.0207432

**Published:** 2018-11-19

**Authors:** Joseph Gibbons, Michael Barton, Elizabeth Brault

**Affiliations:** 1 Department of Sociology, San Diego State University, San Diego, California, United States of America; 2 Department of Sociology, Louisiana State University, Baton Rouge, Louisiana, United States of America; University of Zurich, SWITZERLAND

## Abstract

Gentrification has been argued to contribute to urban inequalities, including those of health disparities. Extant research has yet to conduct a systematic study of gentrification’s relation with neighborhood health outcomes nationally. This gap is addressed in the current study through the utilization of census-tract data from the Center for Disease Control’s 500 Cities project, the 2000 Census and the 2010–2014 American Community Survey to examine how gentrification relates to local self-rated physical health in select cities across the United States. We examine gentrification’s association with neighborhood rates of poor self-rated physical health. We contextualize this relationship by evaluating gentrification’s relation with city-level self-rated health inequalities. We find gentrification was significantly and positively related with self-rated physical neighborhood health outcomes. However, the presence and magnitude of gentrification within a city was not associated with health outcomes for cities overall. Based on these findings, we argue that gentrification’s health benefits for cities are limited at best, though gentrification does not appear to be associated with deepening city-level health inequalities, either.

## Introduction

Gentrification has garnered substantial ubiquity in the national discussion of urban inequality. It is a vivid neighborhood-scale expression of a broader process of growing social and economic inequality that has become systemic in cities over the past half-century [1]. Using Kennedy and Leonard’s [2] definition of gentrification, “the process by which higher income households displace lower income [households] of a neighborhood, changing the essential character and flavor of that neighborhood” (p 5), CDC officials warn gentrification carries implications for the health of neighborhood residents because gentrification is associated with increased personal distress among low income and longstanding residents [3]. Moreover, CDC officials argue the changes a local community experiences due to population turnover weaken local social support infrastructure, exacerbating health issues for residents of areas experiencing gentrification. Further, they conjecture gentrification can have broader implications for cities because displacement associated with gentrification, whether voluntarily or involuntarily, can lead to poorer health outcomes for neighborhoods that receive households leaving gentrifying neighborhoods. While these implications are often discussed, only a few studies directly assessed the relation of gentrification with health outcomes [4–8]. These studies offer at best mixed evidence of a relationship between gentrification with neighborhood health outcomes, with little sense of its overall implications for cities.

Gentrification’s connection with urban health raises several questions and concerns. Despite the concern expressed by the CDC [3], there is reason to suspect gentrification is positively associated with neighborhood health. Indeed, the introduction of new resources and improved conditions associated with gentrification can provide blanket benefits to neighborhoods [2,9,10]. Also, scholarship raised doubts about the significance of gentrification for cities, questioning its severity and proliferation. Several studies noted gentrification typically occurs in only a handful of neighborhoods within a city [1,11,12]. Further, scholars have questioned whether gentrification was independent from other forms of urban inequality such as racial and economic segregation [1]. It has been difficult to determine the full scale of gentrification’s relation to health for a city because previous studies were confined to specific cities or regions. While gentrification research is not limited to large cities, much of the existing research examined neighborhood changes in New York, Chicago and Los Angeles [13,14]. This raises questions about the generalizability of the association of gentrification with health.

Data limitations previously prevented full examination of these questions. The CDC’s 500 Cities project offers non-restricted census-tract and city level health estimates for the 500 largest cities in the United States [15], thereby allowing for the study of the association of gentrification with health outcomes at the neighborhood and city level. The current study uses this data to evaluate the association of gentrification with poor self-rated physical health for neighborhoods within cities across the United States. To understand the scope of gentrification’s association with health, we determine whether the stage of gentrification in each neighborhood was related to poor self-rated physical health. We also look at how the proportion of gentrifying neighborhoods in a city relates to the overall rate of poor self-rated health. Doing this will help us to understand the overall connection of gentrification with health inequalities in a city. In the following sections, we unpack the literature on neighborhood correlates of health and how gentrification fits into the existing discourse.

### Neighborhoods and health

Research suggests several reasons why neighborhood context is important for self-rated health, with poverty playing a prominent role in existing work. For one, low income communities often lack rescources to improve health such as healthy food options, quality healthcare, and park space [16–19]. In addition, disadvantaged neighborhoods often feature greater signs of physical (broken windows or graffiti) or social disorder (public drunkenness or rowdy youth groups), which can exacerbate poor health. Exposure to such environmental hazards was found to be associated with psychological stress or chronic health problems [20–22]. Thus, disadvantaged neighborhoods are more likely to contain environmental risks associated with poor health outcomes. Gentrification, however, often reduces the prevalence of such problems and as such should be associated with lower rates of such environmental risks and in turn better health outcomes. This will be elaborated upon in the next section.

### Gentrification and neighborhood health

Scholars have debated the implications of gentrification for neighborhoods for nearly 60 years with studies finding support for hypotheses that gentrification either improves or harms neighborhoods [14]. An often-discussed benefit of gentrification is the reintroduction of resources to impoverished neighborhoods due to an increase in the proportion of middle and upper-class households. These resources range from better quality food options to the local presence of health clinics [9,23,24] The greater availability of resources associated with this transition should be associated with improvements to quality of life and in turn better self-rated health. In addition, more affluent neighborhoods are strongly associated with social cohesion and networks that improve care [25–28]. This social cohesion can also be converted to improvements in neighborhood resources associated with better quality health [29,30], including some of the new restaurants and clinics discussed above as well as improvements to quality of life like cleaner streets and reductions in crime [9,31–33].

Despite research highlighting the benefits of gentrification for neighborhoods, there is also evidence finding the opposite. Foremost, there is evidence that gentrification can destabilize neighborhoods. Population turnover because of gentrification can weaken local neighborhood communities. This potential has been frequently highlighted in gentrification research discussing the potential for residential displacement [34–37]. Even if displacement is not extensive, the influx of new residents could undermine the existing neighborhood community, weakening the positive health effects. Neighborhoods with large population shifts were found to have poor health outcomes [26,38–40]. As such, even if gentrification increases the affluence of the area, the weakening of local community may be harmful to local health.

A notable critique of previous research on the connection of gentrification with health was its limited incorporation of the importance of neighborhood trajectories. This is important because the decline in stability and resulting harm to neighborhood health may only be short term because gentrification often increased rates of homeownership and in turn improved residential stability [2,9,41]. As such, the stage of gentrification could be of great importance—a neighborhood with more advanced gentrification could have more community and thus better health outcomes [14,42].

To further complicate the importance of gentrification for health, it is important to recognize improvements in gentrified neighborhoods may not be associated with city-wide health inequalities. First, disadvantaged neighborhoods that do not experience gentrification would assumedly continue to have health problems. The selective nature of gentrification thus means that at best, only a few neighborhoods would benefit from the improvements associated with gentrification. Second, the improved neighborhood health associated with gentrification is very likely driven by neighborhood selection of more affluent residents. In other words, gentrifying neighborhoods being healthier is quite possibly due to the preexisting good health of the affluent residents moving into these places. It is then possible that improvements in health at the neighborhood level are the result of population changes, not improvements to the neighborhood. Existing non-restricted data does not allow the distinction of new residents from longstanding residents. Nonetheless, neighborhood level residential changes can be associated with city level changes that can be measured [43,44]. This population shift can have broader effects for other neighborhoods in a city due to displacement.

A substantial body of research has documented the potential for residential displacement in gentrified neighborhoods because the increased affluence in the area makes it more difficult for incumbent households to live in the area [34,36,45]. While little is known about the destinations to which those displaced by gentrification move, findings reported by Ding, Hwang and Divringi [42] using restricted credit data suggest displaced households moved to non-gentrified neighborhoods within the same city. Similarly, eviction research found those forced to find housing with limited options in a city can experience adverse health effects including settling for inferior housing with potentially more health hazards like lead paint in addition to having fewer financial resources to maintain a healthy lifestyle [38,46]. Thus, displacement from gentrification could be related to an aggregate effect of neighborhoods that are less healthy due to the clustered disadvantage outside of the gentrifying neighborhoods. In sum, even if gentrifying neighborhoods prove more healthy than other neighborhoods, cities overall may be less healthy due to the displacement of poorer residents.

The existing gentrification research also continues to debate the intensity of gentrification-related displacement [23,42,44,47]. While there is extensive qualitative evidence to indicate the existence of displacement [9,23,44,48,49], questions remain as for how much gentrification-related displacement impacts cities overall. A recent quantitative study utilizing a national eviction dataset found no relation between gentrification and eviction [50]. This is not to say that the potential for gentrification-related displacement should be ignored, but instead that displacement may be subordinate to broader urban processes shaping cities.

Given the mixed findings of research on the implications of gentrification for neighborhoods and cities overall, it is not surprising that the limited existing research on gentrification and health produced contradictory findings. Gibbons and Barton [4] found gentrification in Philadelphia during the 2000s was modestly associated with better self-rated health, but also that gentrification was associated with poor self-rated health among nonwhite residents who were at the greatest risk of displacement. In contrast, other studies found gentrification was associated with increased rates of mortality [51] and preterm births among residents of color [5] in neighborhoods experiencing gentrification. As such, it remains unclear whether the benefits of gentrification extend to health outcomes and what this means for the non-gentrifying neighborhoods in a city.

Research also questioned how substantial gentrification’s connection with health is compared with other inequalities. For example, Florida [1] recently argued gentrification’s effects on cities and neighborhoods were secondary to deeper issues of economic and racial/ethnic segregation. This ties into longstanding scholarship that points to the deepening divide among already affluent neighborhoods and low-income neighborhoods [1,46,52,53].

### Overview and hypotheses

Despite the ubiquity of gentrification in the broader discussion on urban inequality, there is a lack of conclusive evidence demonstrating gentrification is associated with neighborhood health outcomes. We theorize gentrification may be related with better health in the neighborhoods in which it takes place, but that such benefits would not extrapolate to the city level. Either the gentrification takes place at too small of a volume to have a meaningful impact on a city’s health or neighborhoods which are not gentrifying are experiencing worse outcomes because of displacement, which undercuts the net benefit of gentrification.

The present study examines the association of gentrification with physical health at a neighborhood-level and city-level. In doing so, we test the following hypotheses: Hypothesis A) gentrification is negatively associated with poor self-rated physical health in neighborhoods because gentrification is associated with the reduction in neighborhood disadvantage and the potential exposure to harmful environmental influences. We also theorize gentrification is not associated with the overall health outcomes of a city, which is assessed by testing Hypothesis B) the proportion of gentrifying tracts is not positively associated with the city rates of poor self-rated health. Finally, we believe gentrification’s effects carry over regardless of city size with Hypothesis C) gentrification’s relation to self-rated health at a city and neighborhood level is consistent regardless of city size.

## Data and methods

### Data sources

Our dependent variables were derived from the 500 Cities project, which created census tract-level estimates of the 2014 wave of the Behavioral Risk Factor Surveillance System (BRFSS) for 500 American cities [15]. The BRFSS is a national household telephone survey administered every two years by the CDC and typically reported at the state and county levels [15]. Tract and city estimates from the BRFSS were derived through multilevel strategy linking geocoded county-level BRFSS data to block-level demographic data from the 2010 Census to predict the characteristics of health by location [54]. To validate this method, the CDC created county-level estimates out of their local area estimations and compared them to the raw BRFSS estimates for counties in Missouri [55] and Massachusetts [56]. They found these measures closely paralleled one another. In identifying cities for these estimates, the CDC generally selected census designated places with the ‘city’ designation and at least 66,000 residents as of 2010. To ensure at least one city for each state, the CDC made exceptions to include Honolulu, HI, which is not technically designated as a city, as well as smaller cities like Cheyenne, WY, which had a 2010 population below 66,000. Thus far, tract estimates have only been generated for the 2014 BRFSS data.

Data for our independent variables were collected from the 1990 Census, 2000 Census and the 2010–2014 American Community Survey. To adjust for tract boundary changes between censuses, we obtained 1990 and 2000 Census data from the National Historical Geographic Information System (NHGIS), a service which interpolates pre-2010 census data to 2010 boundaries.

In using census tracts and cities as levels of analysis in a study of gentrification and health limits our ability to directly measure individual health outcomes in these places and to distinguish the health differences between gentrifiers and longstanding residents. While this limits the scope of our argument to aggregate effects, we were unable to locate a nationwide non-restricted individual-level dataset that could be linked to the 500 Cities data. Nonetheless, studying neighborhoods allows us to compare the overall connection of gentrification with self-rated physical health across neighborhoods in many cities. Our city-level analysis assesses whether gentrification has an overall effect on health within a given city.

### Dependent variables

Based on the 500 Cities data, we measure two forms of neighborhood self-rated health. Poor self-rated physical health is derived from the percent of respondents over age 18 who reported fourteen or more days of poor physical health in the past thirty days. While self-rated health is often reported on a Likert scale ranging from very poor health to very good or excellent health, the 500 Cities data instead reports the percentage of those in poor health. This is a common practice in studies that examine the relationship neighborhood characteristics have to poor health, including gentrification [4]. Self-rated health has been widely adopted in health research [57,58]. It is useful not only for its ability to predict disease and mortality [59], but also for its capacity to indicate the general well-being of individuals [60]. One important limitation to the 500 City estimates is that only the crude rates of poor self-rated health are reported, without any normalization or stratification by demographic features including race/ethnicity, income, or age. We model for factors like age, race/ethnicity, and socio-economics but this omission could be a source of bias in our results.

### Independent variables

Our focal neighborhood predictor is gentrification, which has been measured in several ways [11,42]. The current study settled upon the threshold measure devised by Ding et al. [42], which was heavily influenced by the measure developed by Freeman [61] for a few reasons. First, the Freeman [61] measure was developed with a national sample of census tract data, much like the current study. Ding et al.’s [42] measure offers more flexibility than Freeman’s [61] measure by dropping the requirement that gentrifiable tracts contain a lower proportion of housing stock built in the past twenty years than the median for the city. This expectation is more characteristic of older forms of gentrification as well as the gentrification found in East Coast cities. Second, and more importantly, Ding et al. [42] developed an iteration of their measure accounting for stages of gentrification, which we will discuss in greater detail shortly. Third, both Ding et al.’s [42] and Freeman’s [61] measure allow for comparison of gentrified places with those which had the potential, but so far ‘failed’ to gentrify. We used non-gentrifying tracts as our reference group to better gauge the relationship gentrification has with health in neighborhoods.

Replicating the Ding et al. [42] measure, tracts in each city were classified as gentrifiable in 1990 or 2000 if they featured a median household income below that of the city in which they were located in 1990 or 2000, respectively. Eligible tracts were then coded into four dichotomous categories. *Non-gentrifiable* (1) tracts featured a median income at the start of the decade (1990 or 2000) that fell above the citywide median, thus serving as a crude measure of already advantaged places in each city. *Gentrifiable* (2) tracts featured a decade-start median household value below the median value reported for the city within each tract. A tract was deemed *gentrifying* (3) if it was gentrifiable at the start of a decade and experienced an increase in gross rent or median home value above the citywide median and an increase in college-educated residents above the citywide median between the start and the end of a given decade (1990–2000 or 2000–2014). *Non-gentrified* (4) tracts were gentrifiable, but which failed to meet the criteria of gentrifying (reference group).

Given Brown-Saracino’s [14] recent call for more attention to the importance of the stage of gentrification, we also incorporated stage measures of gentrification. To do this, we used the strategy described by Ding et al. [42] to determine whether tracts had experienced gentrification during the 1990s and 2000s. Tracts that were gentrifiable in 1990 and experienced gentrification during the 1990s but were no longer gentrifiable in 2000 were classified as old gentrification. Tracts that experienced gentrification during the 2000s, but not during the 1990s were classified as recent gentrification. Tracts that experienced gentrification during both the 1990s and 2000s were classified as continued gentrification. Tracts that were gentrifiable in 1990 and 2000 but did not experience gentrification were classified as never gentrified. These categories are mutually exclusive, reflecting all the measurable changes associated with gentrification in these tracts from 1990–2014.

With the 2010–2014 American Community Survey we also account for other neighborhood-level factors found to be important for self-rated health. This includes measures of race/ethnicity: percent Black, percent Asian, percent Hispanic to determine how independent gentrification’s effects are from racial/ethnic composition. Neighborhood racial/ethnic composition has an important association with local self-rated health, with nonwhite neighborhoods having a greater likelihood of experiencing worse overall health outcomes than other kinds of neighborhoods [62]. We also measure neighborhood stability: including percent of the population living in the same house for at least five years and percent home owner-occupied, given the association of stability with health [26,38–40]. This measurement allows us to determine how independent the socio-economic changes of gentrification are from the broader change of new residents moving into a neighborhood. We include a measure of the median age of the population in a tract to determine the potential relation of age to health given age was found to be positively associated with poor health [63]. We account for socio-economic disadvantage with the unemployment rate because of the association of lack of employment with poor health [63]. These variables are reported both at a tract level for the tract-level analysis and aggregated for the city-level analysis.

We also account for how much gentrification’s relation with neighborhood health can be explained by the broader effects of city-level inequality. First, we measure city-level economic inequality with the Gini coefficient, which measures the unequal distribution of income by tract. Second, we measure city racial/ethnic segregation, which is a powerful predictor of health disparities in cities [64]. We use the Information Theory Index to estimate multi-group dissimilarity for the White, Black, Hispanic and Asian populations for each city [65]. These measures are treated as the level two measures in our multilevel models and as regular predictors in our city-level models. We discuss our measurement strategy more in the next section.

Our estimations distinguish large cities from the rest of the sample of cities because much of the gentrification literature has focused on larger cities [13,14]. Our goal in this strategy is to determine whether gentrification has a unique relation with health in large cities compared to smaller cities. In determining large cities, we select those cities with populations at or above the 75 quartile of the 500 sampled cities. The largest of these cities was New York, NY, with a population of 8,175,133 in 2010 and the smallest was the consolidated city-county of Honolulu, HI, with a population of 953,207 in 2010.

### Estimation strategy

We employed two analyses, a multilevel model which includes neighborhoods nested into their respective cities and an OLS analysis focused just on city-level effects. The Level One units in our multilevel analyses are census tracts, while the Level Two units are cities. Our Level Two analyses of the multilevel models allow us to explore the broader effects of gentrification by situating gentrifying and non-gentrifying neighborhoods within their larger city context [66]. All continuous variables are grand-mean centered. We used the technique described by Clogg et al. [67] to determine whether the differences in the gentrifying coefficients were significantly different between models. We recognize analyzing aggregate measures such as these limits our ability to make claims about individual level outcomes because of the potential for ecological fallacy and the modifiable areal unit problem [68,69]. We are cautious in the interpretation of our result to keep our arguments and assumptions reserved to group-level effects.

## Results

### Descriptive results

Descriptive results are presented in Table 1. Just under 13 percent of respondents in the average tract reported experiencing poor self-rated physical health. Given the importance of the assessment of different stages of gentrification, we decompose our gentrification measure into four categorical variables. About 44 percent of tracts were non-gentrifiable. Another 10 percent of tracts were classified as old gentrified, suggesting gentrification either stalled or was completed in these tracts. Statistics for the continued gentrification show about 4.3 percent of tracts were classified as having experienced gentrification throughout the 1990s and 2000s. The statistics for the recent gentrification category show about 14 percent of tracts were classified as having experienced gentrification only during the 2000s.

**Table 1:** Tract level descriptive statistics.

Variables	Values
Low Self-Rated Physical Health	12.998 (4.538)
Non Gentrifiable	44.400 (49.700)
Old Gentrification	10.200 (30.300)
Continued Gentrification	4.300 (20.200)
Recent Gentrification	14.200 (34.900)
Percent White	45.544 (29.793)
Percent Black	20.280 (27.195)
Percent Asian	7.120 (11.278)
Percent Hispanic	23.633 (24.912)
Percent Living in Same House	29.900 (12.867)
Unemployment Rate	6.914 (4.003)
Percent Owner Occupied	51.488 (24.188)
Median Age	35.729 (7.137)
Theil’s H	0.217 (0.118)
Gini	0.235 (0.051)
South	30.540 (46.060)
West	32.440 (46.815)
Midwest	21.010 (40.741)
Northeast	19.000 (39.000)
Tract Was in a Large City	2.239 (15.301)

N = 29305

Standard Deviation in parentheses.

Turning to the other controls, Whites constituted the largest racial group in these cities (45%), followed by Hispanics (24%), Blacks (20.2%), and Asians (7.4). About 50 percent of residents in the average tract owned their homes and 30 percent lived in the same house for at least five years in both samples. The median age was about 35 years old. The score for the Theil’s H for the average neighborhood’s city is 0.217 and the Gini Coefficient is 0.235. This suggests the average neighborhood is in a moderately segregated city. Though it should be noted that these scores do not account for potential regional level inequality. Statistics for the regional variables show about 30 percent of tracts in each sample were in the South, about 31 percent of tracts were in the West and about 21 percent of tracts were located in the Midwest. Only 2.3 percent of the tracts in this study were located within large cities.

Our analyses also examine the importance of each of the gentrification stages for poor self-rated physical health at the city level. Table 2 presents descriptive statistics for these analyses. Given the general similarity of these results to the tract-level results, they are not discussed in detail here.

**Table 2:** City level descriptive statistics.

Variables	Values
Low Self-Rated Physical Health	12.333 (2.61)
Low Self-Rated Mental Health	12.306 (2.214)
Non Gentrifiable	44.100 (5.600)
Old Gentrification	10.800 (6.100)
Continued Gentrification	3.400 (3.600)
Recent Gentrification	13.700 (6.200)
Percent White	48.700 (25.300)
Percent Black	15.000 (22.900)
Percent Asian	8.600 (15.400)
Percent Hispanic	27.700 (36.400)
Percent Living in Same House	13.100 (9.100)
Unemployment Rate	5.800 (4.200)
Percent Owner Occupied	26.200 (19.900)
Median Age	36.881 (7.896)
Theil’s H	0.136 (0.083)
Gini	0.203 (0.055)
South	30.400 (46.000)
West	39.000 (48.800)
Midwest	19.800 (39.900)
Northeast	10.800 (31.100)
Large City	2.400 (15.320)

N = 500

Standard Deviation in parentheses

### Multilevel results

The results of our multilevel analyses of physical health are presented in Table 3. All predictors were standardized to facilitate comparison of the results across models. We utilized a stepwise strategy to determine the unique associations of our gentrification measures across our models. Model 1 presents the gentrification results alone, Model 2 has the other tract-level controls alone, Model 3 combines tract-level results and Model 4 adds the level two variables.

**Table 3:** Results of self-rated physical health multilevel analyses.

	1	2	3	4
**Level 1 (N = 29305)**				
Old Gentrification	-2.408***		-0.799***	-0.800***
	(0.072)		(0.049)	(0.049)
Continued Gentrification	-3.477***		-1.397***	-1.400***
	(0.101)		(0.070)	(0.070)
Recent Gentrification	-1.268***		-0.680***	-0.680***
	(0.064)		(0.043)	(0.043)
Non Gentrifiable	-5.195***		-2.156***	-2.161***
	(0.047)		(0.040)	(0.040)
Percent Black		1.839***	1.631***	1.626***
		(0.024)	(0.023)	(0.023)
Percent Asian		-0.017	-0.008	-0.008
		(0.020)	(0.019)	(0.019)
Percent Hispanic		2.464***	2.136***	2.134***
		(0.027)	(0.026)	(0.026)
Percent Living in Same House		-0.911***	-0.962***	-0.965***
		(0.022)	(0.021)	(0.021)
Unemployment Rate		1.038***	0.907***	0.907***
		(0.020)	(0.019)	(0.019)
Percent Owner Occupied		-1.878***	-1.451***	-1.451***
		(0.029)	(0.029)	(0.029)
Median Age		1.077***	0.968***	0.968***
		(0.021)	(0.020)	(0.020)
**Level 2 (N = 500)**				
Theil’s H				0.269*
				(0.162)
Gini				0.305***
				(0.106)
South		0.575*	0.254	0.148
		(0.337)	(0.324)	(0.318)
West		-0.325	-0.571*	-0.282
		(0.328)	(0.314)	(0.316)
Midwest		1.707***	1.157***	1.116***
		(0.360)	(0.345)	(0.339)
Large City				-0.092
				(0.086)
Constant	15.411***	12.894***	14.253***	14.568***
	(0.126)	(0.290)	(0.279)	(0.284)
Observations	26,620	26,620	26,620	26,620
AIC	138,767.20	120,245.00	117,390.20	117,381.300

Standard Errors in Parentheses;

*** p < 0.001,

** p < 0.01,

* p < .05;

Bolded gentrification coefficients are statistically significantly different from Model 1 (P < .001).

In Model 1, we see that all forms of gentrification have a significant and negative relation with poor self-rated health. This supports Hypothesis A. The magnitude of these coefficients declines with the introduction of the controls in Models 3 and 4. For example the strength of the coefficient for Old Gentrification declines from -2.408*** in Model 1 to -0.800*** in Model 4. These declines were statistically significant, which indicates other neighborhood processes were important for rates of poor self-rated health, but the coefficients for each of the gentrification stages remained highly significant. There is also some variation in the magnitude of gentrification’s association with poor health depending on its stage. Tracts that experienced continued gentrification were the most likely to feature lower rates of poor self-rated health (-1.399***), but the coefficients for old gentrification (-0.800**) and recent gentrification (-0.680***) indicate these neighborhoods were also less likely to report poor health. While the results suggest health advantage for gentrifying tracts, a stronger health advantage can be found in non gentrifiable tracts, which in Model 4 had a coefficient of -2.161***.

The results for the control variables were mostly consistent with previous research on neighborhood correlates of health. The magnitude of these results changes little from their introduction in Model 2 with the addition of the gentrification variables in Model 3 or the Level Two variables in Model 4. As such, for the following we report the Model 4 results. Tracts that featured larger Black (1.626***) and Hispanic (2.134***) populations were more likely to report higher rates of poor self-rated health, but the percent of Asian residents in a tract was not a significant predictor of poor self-rated health. Tracts characterized by lower rates of residential mobility (-0.965***) and higher rates of homeownership (-1.451***) were more likely to feature lower rates of poor self-rated health. Tracts with greater rates of unemployment (0.907***) were more likely feature higher rates of poor self-rated health. The median age of tracts was positively associated with rates of lower self-rated health (0.968***). The coefficient for Theil’s H (0.269*) indicates rates of poor self-rated health were higher in tracts characterized by greater racial segregation. Similarly, the coefficient for the Gini variable (0.305***) indicates rates of poor self-rated health were higher in economically disadvantaged cities. The coefficient for the large cities variable was not significant. Specifically, this indicates rates of poor self-rated health were not significantly different between large cities and the smaller cities. This null finding essentially supports Hypothesis C, that gentrification’s effects should carry regardless of city size. Results for the region variables did indicate rates of poor self-rated health in tracts located in the Midwest (1.116***) were significantly higher than tracts located in the Northeast but did not identify significant differences with tracts located in the South or West.

### City level analyses

Given the apparent health boost of gentrification to neighborhoods, it is not surprising cities have frequently looked to gentrification to address the issues of disadvantaged neighborhoods [1]. Its positive effect, however, is questionable. First, gentrification may trigger displacement which could be associated with more poor health in non-gentrifying neighborhoods, depreciating a city’s health overall. Second, the positive health in gentrifying neighborhoods could just be the movement of more affluent already healthy residents into these communities. This could limit gentrification’s association with health. Third, gentrification is often limited to part of a neighborhood or a few neighborhoods within a city, which may mean any connection of gentrification with self-rated health is masked at the city level [9,70]. Based upon this logic, Hypothesis B predicted the proportion of gentrifying tracts in a city would not be associated with the city rates of poor self-rated physical in cities. Relatedly, Hypothesis C predicted gentrification’s relation to self-rated health at a city and neighborhood level would be consistent regardless of city size.

The city-level analysis, reported in Table 4, largely finds support for Hypotheses B and C. The results of these measures suggest the proportion of gentrification does not have an association with city-level health. None of the measures of gentrification were associated with city-level rates of low self-rated physical health. Taken together, the results of this table provide very limited evidence for claims that gentrification has a large impact on cities.

**Table 4:** Results of self-rated physical health city level analyses.

Variables	Coefficients
Proportion Old Gentrifying Tracts	0.123 (0.111)
Proportion Continued Gentrifying Tracts	-0.030 (0.099)
Proportion Recent Gentrifying Tracts	-0.080 (0.104)
Proportion Non Gentrifiable	-0.031 (0.109)
Percent Black	0.150 (0.125)
Percent Asian	-0.412*** (0.108)
Percent Hispanic	0.742*** (0.124)
Percent Living in Same House	-0.349*** (0.113)
Unemployment Rate	0.501*** (0.141)
Percent Owner Occupied	-0.497*** (0.122)
Median Age	0.228* (0.118)
Theil’s H	1.083*** (0.135)
Gini	-0.066 (0.124)
South	-0.776* (0.356)
West	-0.329 (0.344)
Midwest	-0.802* (0.379)
Large City	-0.309* (0.102)
Constant	12.859*** (0.298)
N	500
R^2^	0.359

Robust standard errors clustered by city used; Standard errors in parentheses.

*** p < 0.001.

**p < .01.

* p < .05.

## Discussion

An extensive body of research has examined the importance of neighborhoods for health with research repeatedly finding disadvantaged neighborhoods have higher rates of poor health outcomes [4,18,20,21,38,62,71]. An important limitation of previous research was that few of these studies examined the importance of gentrification for health. While there is much concern surrounding gentrification’s role in the deepening social and economic gulf found in cities, existing research has been divided as for how gentrification relates to health in cities [4,5,7,8]. Findings of the current study indicate residents of neighborhoods experiencing gentrification reported overall better physical health outcomes than those living in neighborhoods that had not experienced gentrification, regardless of its stage.

These findings stand out for their robustness. All of the previous research on gentrification and health was limited to case studies of specific cities, namely Montreal [8], New York City [5], Philadelphia [4], and metropolitan areas in California [6]. While each of these studies analyzed variation in health outcomes with large samples of neighborhoods, all were potentially limited in their generalizability because they focused on at most a few large cities or regions. A similar critique has been made of the broader research on the association of gentrification with other outcomes [1,14]. The current study overcame this limitation by utilizing a unique dataset that included census tracts from 500 cities from across the United States. In addition to the sheer number of cities and census tracts represented in the data, the current study examined the association of gentrification with health outcomes in a variety of cities, not just larger cities. We found the neighborhood-level association of gentrification with better physical health outcomes holds regardless of city size or regional location, meaning gentrification’s relation to neighborhoods is largely consistent.

These findings are also important because they broaden the conversation of gentrification beyond select neighborhoods or cities. For one, we found the association of gentrification with city level health was more limited compared to what we saw at the neighborhood level. This is not surprising given that gentrification is often limited to a small section of a single neighborhood or to a few neighborhoods within a city [9,14,70], but is somewhat problematic given that cities have increasingly looked to gentrification as a means of addressing social problems [1]. The presence and proliferation of gentrifying census tracts within a city was not associated with variation in poor self-rated physical health for that city overall. In other words, gentrification does not appear to be associated with urban health inequality. However, gentrification is not leading to net improvements for cities, either. Any improvements gentrification offers are instead highly localized to the neighborhoods within which it is taking place. Further, it is important to recognize gentrification is associated with changes in the class and racial composition of neighborhood residents in addition to features of the physical space such as changes in local stores and improvements to local infrastructure that were unable to be assessed in the current study. These results call us to reconsider gentrification’s place in the broader discussion of urban inequality relative to other forces of inequality at a neighborhood level.

This is not to say gentrification has unilaterally positive implications for residents of gentrifying neighborhoods or no implications for health issues at the city level. As the filmmaker Spike Lee once argued, gentrification affects people, not neighborhoods [1]. Our findings do not tell us how the individual residents of these neighborhoods responded to gentrification. That physical health outcomes tended to be better in gentrified neighborhoods supports arguments that gentrification may introduce resources to disadvantaged neighborhoods that result in improvements to quality of life [9,23,24]. However, the tide does not always raise all boats. While any direct inference at the individual level with our results would constitute ecological fallacy, we suspect the relationship of gentrification with health at the tract level is related more to the presence of affluent residents in these places as opposed to all residents, rich and poor, reporting better health. This suspicion is supported by Gibbons and Barton’s [4] study of gentrification and self-rated health in Philadelphia, which found that while gentrifying neighborhoods overall had a modest positive relation with health, nonwhite residents in these neighborhoods were more likely to report poor self-rated health. This echoes findings of the broader gentrification literature which suggests gentrifiers and incumbent residents often differ in their perceptions of neighborhood changes [34,45]. Consequentially, our analyses could be overlooking individual health problems within these neighborhoods. As such, the new resources in gentrifying neighborhoods may have little to do with the health in these neighborhoods. In addition, most scholarship on gentrification agrees that the lack of access to these neighborhoods for low income and nonwhite populations presents an important problem. Residents who cannot afford to live in gentrifying neighborhoods must instead live in neighborhoods with worse potential health outcomes. Future work will need to synthesize larger neighborhood level processes across cities with those taking place at an individual level to better unpack this dynamic.

There are several other limitations with our data and methods that need to be addressed in future research. The 500 Cities data’s coverage of suburbs is inconsistent given the focus on places designated as cities, meaning it cannot capture metropolitan effects. The clustering of wealth and poverty varies from region to region with economic advantage often favoring suburbs over cities, but this is not unilateral nationwide [1]. Further, our analyses potentially exclude people displaced from central cities to peripheral suburbs because of gentrification. This displacement could present substantial health disparities. Our analyses thus miss out on the full potential variation of haves versus have-nots. Also, we are not able to directly measure whether gentrification is associated with changes in neighborhood health over time because the 500 Cities data only includes a single cross-section. Finally, this study only analyzed residential gentrification. It is not clear how gentrification of local commerce may have mediated or even moderated the relationships we found.

As inequalities in the United States deepen, problems in health only stand to grow. While gentrification does not appear to have a major influence on city health inequalities, it does not have a meaningful role in reducing these inequalities either. At best, gentrification is an unsatisfactory solution to city health problems, with benefits confined to specific neighborhoods that may not benefit all the residents within. Nonetheless, gentrification is not likely to stop soon. As Schlichtman and co-authors noted recently [72], it is worthwhile for scholarship to explore how gentrification can be made beneficial to all in a city. The introduction of affluent residents and resources has been argued in other contexts to be a solution to urban health disparities [73]. For the benefits of gentrification to be realized, however, substantial steps would have to be taken to ensure these neighborhoods and their resources remain accessible to all residents. Accessible housing is a key part of this assurance, whether it be through policy initiatives for affordable housing or affordances to housing costs. This may help assuage concerns about displacement. Gentrification has a reputation as a local incarnation of broader city-level inequalities, but it has the potential to be modified in such a way to be of use for disadvantaged neighborhoods. The challenges of enacting sound equity policies are great, but with the vast problems cities face as the gap between the haves and have-nots widen, such an effort is a worthwhile endeavor.
